# *In vivo* quantitative photoacoustic monitoring of corticosteroid-induced vasoconstriction

**DOI:** 10.1117/1.JBO.28.8.082805

**Published:** 2023-02-24

**Authors:** Donggyu Kim, Joongho Ahn, Eunwoo Park, Jin Young Kim, Chulhong Kim

**Affiliations:** Pohang University of Science and Technology, Departments of Electrical Engineering, Convergence IT Engineering, Mechanical Engineering, Medical Science and Engineering, and Medical Device Innovation Center Group, Pohang, Republic of Korea

**Keywords:** photoacoustic imaging, optical-resolution photoacoustic microscopy, corticosteroid, vasoconstriction, skin anatomy

## Abstract

**Significance:**

Corticosteroids—commonly prescribed medications for skin diseases—inhibit the secretion of vasodilators, such as prostaglandin, thereby exerting anti-inflammatory action by constricting capillaries in the dermis. The effectiveness of corticosteroids is determined by the degree of vasoconstriction followed by skin whitening, namely, the blanching effect. However, the current method of observing the blanching effect indirectly evaluates the effects of corticosteroids.

**Aim:**

In this study, we employed optical-resolution photoacoustic (PA) microscopy (OR-PAM) to directly visualize the blood vessels and quantitatively evaluate vasoconstriction.

**Approach:**

Using OR-PAM, the vascular density in mice skin was monitored for 60 min after performing each experimental procedure for four groups, and the vasoconstriction was quantified. Volumetric PA data were segmented into the papillary dermis, reticular dermis, and hypodermis based on the vascular characteristics obtained through OR-PAM. The vasoconstrictive effect of each skin layer was quantified according to the dermatological treatment method.

**Results:**

In the case of corticosteroid topical application, vasoconstriction was observed in the papillary (56.4±10.9%) and reticular (45.1±4.71%) dermis. For corticosteroid subcutaneous injection, constriction was observed solely in the reticular (49.5±9.35%) dermis. In contrast, no vasoconstrictions were observed with nonsteroidal topical application.

**Conclusions:**

Our results indicate that OR-PAM can quantitatively monitor the vasoconstriction induced by corticosteroids, thereby validating OR-PAMs potential as a practical evaluation tool for predicting the effectiveness of corticosteroids in dermatology.

## 1 Introduction

Following their discovery in the 1950s, corticosteroids have been widely used for inflammatory skin disease.^1^ As anti-inflammatory agents that generally reduce inflammation and immune response, their therapeutic effectiveness and efficacy in treating various skin diseases, such as atopic dermatitis, asthma, and rheumatoid arthritis, have been clinically verified,^2^^–^^4^ and currently, these agents are prescribed for the treatment of skin diseases. The corticosteroid acts in the body through the following mechanism: vasoconstriction, anti-inflammatory, immunosuppressive, and anti-proliferative responses.^5^ For example, the corticosteroid reduces erythema by constricting the capillaries in the shallow dermis through the inhibition of the vasodilators’ secretion (e.g., prostaglandin), thereby exhibiting anti-inflammatory action.^6^^,^^7^ This vasoconstrictor ability—the ability to whiten the skin—determines its effectiveness. Therefore, unlike drugs in other therapeutic classes, such as antifungal and antibacterial agents, topical corticosteroid products are graded and classified according to their effectiveness. However, skin whitening, i.e., the blanching effect, is an indirect indicator of the extent to which corticosteroids cause vasoconstriction, and deviations may arise depending on the difference in skin thickness between individuals.

Notably, the effectiveness of corticosteroids can be assessed by quantifying the mechanism through which they constrict capillaries and eventually whiten the skin; thus, several techniques have been attempted to characterize the blanching effect more accurately. Initially, reflectance spectrometry, laser-Doppler velocimetry, and thermography have been employed and refined for the assessment of the effectiveness; however, these techniques were insensitive, difficult to implement, and less precise for quantitative appraisal.^8^^,^^9^ Recently, colorimetry of the skin-blanching response via a portable chromameter has emerged as a frequently used strategy in medical fields for tracking the progress of the blanching effect.^10^^,^^11^ However, this colorimetric-based vasoconstrictor assay is entirely based on visual evaluation, and it is still difficult to standardize due to inaccurate quantification.^12^^,^^13^ Additionally, deviations may arise due to differences in skin color for each patient. Above all, a majority of the existing methods have indirectly quantified the blanching effect caused by vasoconstriction rather than characterizing the vasoconstriction itself. Thus, the direct quantitative monitoring of vascular changes has not yet been attempted.

Photoacoustic (PA) imaging—a specialized tool for visualizing blood vessels—is a promising candidate for assessing vasoconstriction. PA imaging is a hybrid imaging technique that detects ultrasound (US) waves generated through instantaneous heating and thermal expansion by irradiating optical absorbers with a pulsed laser. In particular, the hemoglobin contained in blood is an endogenous chromophore that is more sensitive than other biological tissues in visible wavelengths; thus, PA imaging can noninvasively visualize the blood vessels without the need for any contrast agent.^14^^–^^16^ In addition, owing to the advantageousness of the three-dimensional (3D) data acquired through PA imaging, this technique is widely used for quantitative volumetric analysis in diverse clinical fields.^17^^–^^22^ Among the currently available systems for PA imaging, PA microscopy (PAM) yields high-resolution images with a resolution of a few micrometers by tightly focusing light and/or acoustic waves on a single spot.^23^^–^^36^ Furthermore, the PAMs have achieved high-speed capability due to the introduction of micro-electro-mechanical system (MEMS)-based mirror scanning,^37^ and since then, various high-speed water-immersible scanners such as galvanometers and polygon-mirror scanners have been developed to increase the imaging speed of PAMs.^38^^,^^39^ Notably, this imaging technique has been used to visualize and monitor microvessels in various studies focusing on wound healing, stimulus response, drug delivery, and regenerative medicines.^40^^–^^44^

In this study, we propose a new approach to assess the vascular effects of corticosteroids using PA imaging. We used an optical-resolution PAM (OR-PAM) system in which optical focusing is much tighter than acoustic focusing because this system has better spatial resolution than acoustic-resolution PAM (AR-PAM) within the optical diffusion limit, allowing us to observe capillary changes with high resolution.^45^ The PA blood vessel images were obtained from a total of 24 mice, six from each of the following groups: (1) corticosteroid subcutaneous injection, (2) corticosteroid topical application, (3) nonsteroidal topical application, and (4) no injection or topical application. We longitudinally monitored vasoconstriction induced by the corticosteroids, quantified the extent of vasoconstriction depending on the skin layers, and compared the changes in blood vessel density between the four groups. Considering the remarkable results obtained via observing and quantifying the corticosteroids-induced vasoconstriction in the skin, we believe that high-resolution PAM has great potential for evaluating the effectiveness of corticosteroids.

## 2 Methods

### 2.1 Optical-resolution Photoacoustic Microscopy

**Fig. 1:**
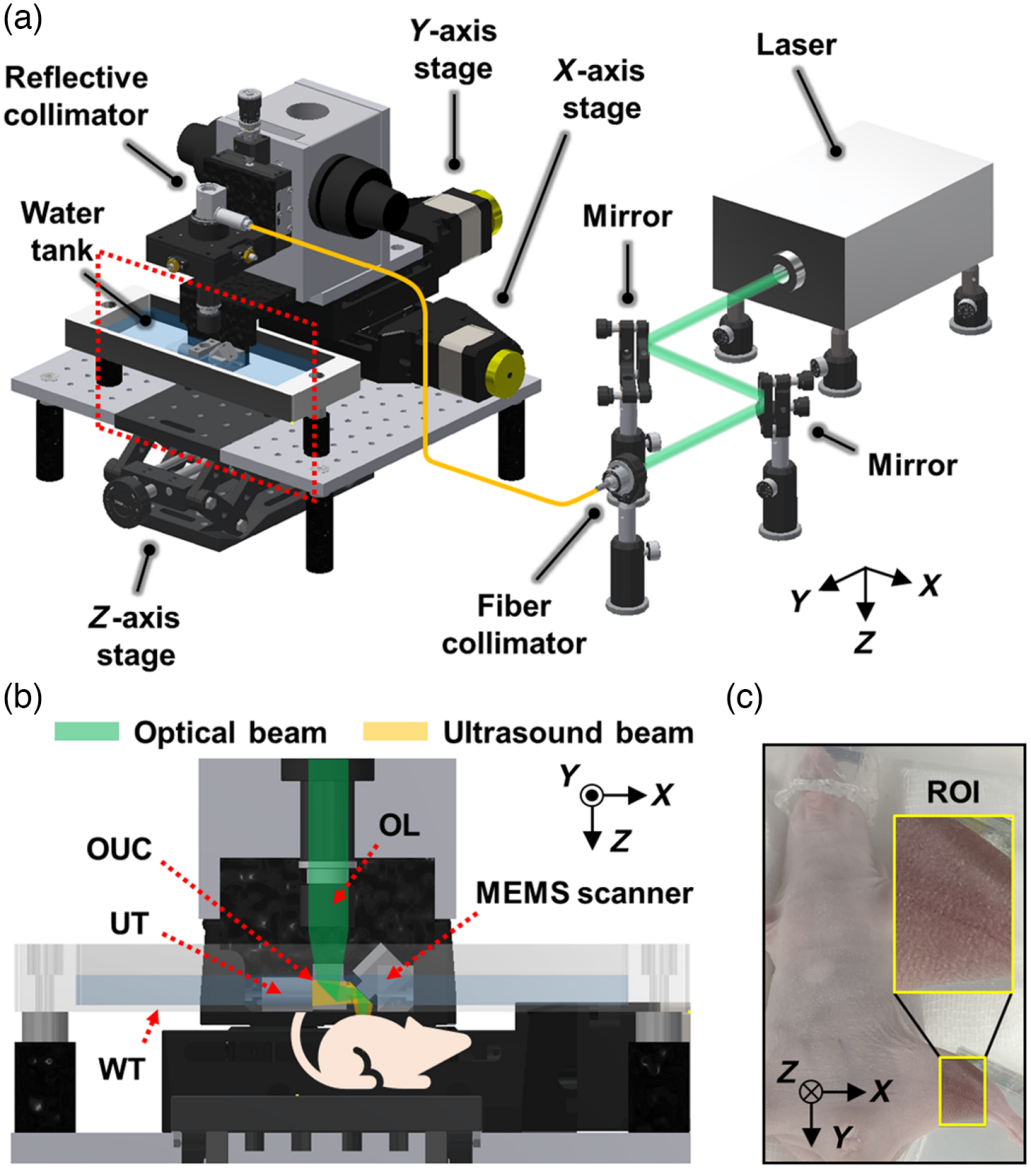
(a) Graphical representation of the optical-resolution PA microscopy (OR-PAM) system based on a micro-electro-mechanical system scanner. (b) Optical and US beam paths in the magnified view of the red dashed box in (a). (c) A mouse mounted on the z-axis stage. The yellow box indicates the region of interest (ROI). OL, objective lens; OUC, opto-ultrasound beam combiner; WT, water tank; UT, ultrasound transducer; and MEMS, micro-electro-mechanical system.

Figure 1(a) shows the OR-PAM system. The system is equipped with a 532-nm nanosecond pulsed laser (AWAVE532-1W-10K, Advanced Optowave, New York) for optical excitation. The laser spot size of our OR-PAM system is 5  μm, and the laser repetition rate for imaging is 10 kHz. The laser beam is delivered to the PAM system using a single-mode fiber (P1-460B-FC-1, Thorlabs, New Jersey). The single-mode beam from the fiber is collimated using a reflective collimator (RC08FC-P01, Thorlabs, New Jersey) and is focused under the skin surface via an objective lens (AC127-050-A, Thorlabs, New Jersey) and a water-immersible MEMSscanning module (OptichoM-MS, Opticho, Republic of Korea). In the scanning module, the laser beam is reflected by an opto-ultrasound beam combiner (OUC) and a MEMS scanner to irradiate the imaging target, thereby generating PA waves [Fig. 1(b)]. The PA waves generated at the target pass through the OUC and are subsequently acquired using a US transducer (V214-BC-RM, Olympus NDT, Massachusetts), which is attached to the beam combiner to convert the PA waves into PA electrical signals. The acquired PA signals are amplified using a 50-dB amplifier (PE15A1013, Pasternack Enterprises, California). The amplified signals are digitized and thereafter transferred to a personal computer using a digitizer (ATS9350, Alazar Technologies, Quebec, Canada) at a sampling rate of 500  MS/s. Additionally, the aforementioned components are installed on stacked motorized linear stages (L-509, Physik Instruments, Germany) to expand the field of view. A z-axis manual stage for the target is utilized to adjust the distance between the system and target for focus optimization. All 3D data were post-processed and analyzed through MATLAB R2021a (MathWorks, Massachusetts) and 3D Photoacoustic Visualization Studio (3D PHOVIS).^46^ The lateral and axial resolutions of the OR-PAM system are 5 and 30  μm, respectively.^47^ The maximum penetration depth of the OR-PAM system is ∼1  mm from the epidermis, and the temporal resolutions for B-scan and volumetric imaging are 80 ms and 48 s.

### 2.2 Animal Preparation and Group Categorization

All experimental procedures for in vivo experiments were approved by the Institutional Animal Care and Use Committee of the Pohang University of Science and Technology. To observe the vasoconstrictive effect of corticosteroids, 24 six-week-old nude mice were categorized into four groups as follows:

1. Corticosteroid subcutaneous injection featuring 0.03% hydrocortisone solution mixed with hydrocortisone powder (H3160-10G, Sigma-Aldrich) and phosphate-buffered saline.
2. Corticosteroid topical application of 2.5% hydrocortisone lotion (Cordicare, DongKoo Bio&Pharma, Republic of Korea).
3. Nonsteroidal topical application of dexpanthenol ointment (Bepanthen ointment, GP Grenzach Produktions GmbH, Germany).
4. No injection or topical application (control).

### 2.3 Experimental Procedure for *in Vivo* PA Monitoring

A mouse was initially anesthetized with a gas mixture of 2.5% isoflurane at a flow rate of 1 l/min using an isoflurane vaporizer (Matrx VIP 3000, Midmark). During the experiment, a mouse in the supine position was anesthetized with 1.5% isoflurane, and its left leg was placed onto a customized leg holder affixed onto the z-axis stage [Fig. 1(c)]. Subsequently, the following procedures were performed for each group:

1. Hydrocortisone subcutaneous injection: 0.05-ml hydrocortisone solution was subcutaneously injected into the thigh of the hind limb using a 0.3-ml insulin syringe (328822, Becton, Dickinson and Company, New Jersey).
2. Hydrocortisone topical application: after applying 0.1 fingertip unit of the lotion, it was allowed to sufficiently absorb into the mouse skin for 10 min, and the remaining lotion was wiped off.
3. Nonsteroidal topical application: the procedure was the same as that applied to group (2) except for the type of lotion.
4. Control group: no injection or topical application was administered.

The amount of hydrocortisone used for groups (1) and (2) was calculated in proportion to the body weight of the six-week-old nude mice compared to the amount of hydrocortisone used in humans in clinical practice.^48^^–^^51^ Additionally, for hydrocortisone subcutaneous injection, the amount of solution was experimentally optimized in both aspects. First, PBS should sufficiently dissolve the hydrocortisone, and second, it should not exceed an excessive amount that causes the mouse’s thigh to swell and interfere with image acquisition. After the procedures for each group, the mouse’s thigh was covered with US gel and subsequently contacted with a thin plastic membrane under a water tank to smoothly propagate the PA waves. The scanning module was submerged in water to ensure that the preset confocal point was focused on the vascular networks in the thigh. Thereafter, PA 3D data were continuously acquired at a time interval of 10 min for 60 min to monitor the changes in vascular density. The laser energy measured on the skin surface was $10\;\mathrm{mJ}/{\mathrm{cm}}^{2}$, which was less than the maximum permissible skin exposure ($20\;\mathrm{mJ}/{\mathrm{cm}}^{2}$) specified by the American National Standards Institute. In addition, the biosafety of our experimental procedure involving laser irradiation after topical steroid treatment has been validated by clinical studies of patients applying topical steroid treatment using clinical PA imaging systems.^52^^,^^53^

## 3 Results and Discussion

### 3.1 Layer-by-layer Vascular Representation from Volumetric PA Data

**Fig. 2:**
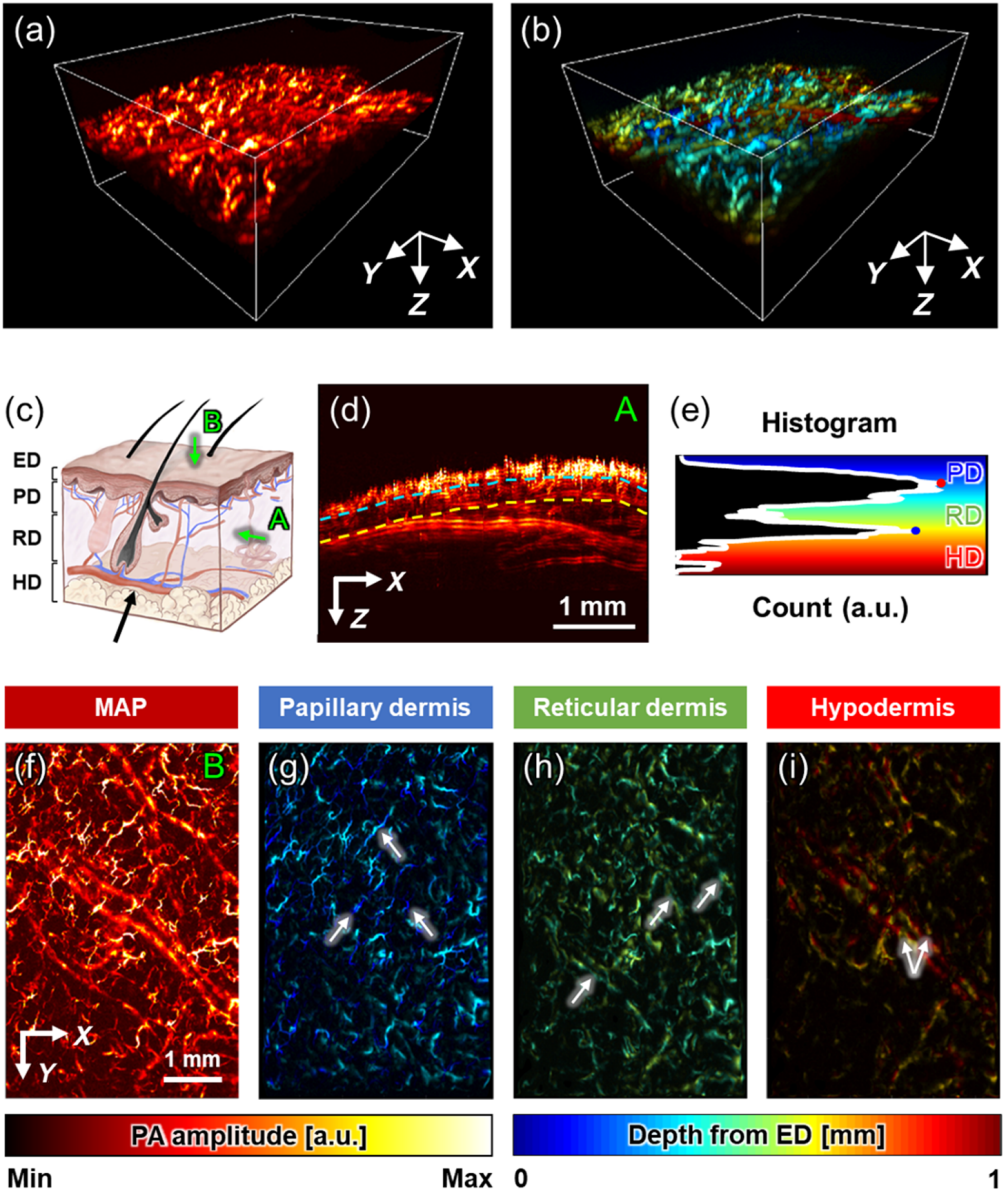
3D-rendered (a) PA and (b) depth-coded views from the ROI in Fig. 1(c). (c) Skin anatomy comprising the ED, PD, RD, and HD layers and vascular networks distributed differently in each skin layer. (d) PA MAP images along the Y direction, which corresponds to the “A” view depicted in (c). (e) Histogram representing vascular distribution depending on skin depth. The count of the histogram represents the number of pixels indicating blood vessels. (f) PA MAP images along the Z direction, corresponding to the “B” view in (c). (g)–(i) Depth-encoded PA MAP images corresponding to the PD, RD, and HD, which is separated with the red and blue dots in (f).

Volumetric PA data (4  mm×6  mm×3  mm along the X, Y, and Z axes at 5-, 10-, and 3-μm intervals, respectively) were acquired at the site of the mouse thigh. Figures 2(a) and 2(b) illustrate the 3D-rendered PA amplitude and depth-encoded images, which render a clear representation of the blood vessels and their networks owing to the higher optical absorption in hemoglobin than in other biological tissues. The PA data were segmented to characterize the skin layers where the administered corticosteroids dominantly acted upon the blood vessels. Generally, the skin layers comprise the epidermis, papillary dermis, reticular dermis (RD), and hypodermis (HD) along the skin depth, and the blood vessels are distributed differently depending on the skin layer [Fig. 2(c)]. Although the epidermis does not contain blood vessels, the capillary blood vessels are densely distributed in the papillary dermis, and relatively large blood vessels with arteriole–venule pairs are present in the HD.^54^^–^^58^ Moreover, the RD includes small blood vessels vertically connecting the blood vessels in the papillary dermis (PD) and HD.^59^ Observably, these features are represented in both the PA maximum amplitude projection (MAP) image along the Y direction [e.g., “A” view shown in Fig. 2(c)] and its depth-dependent histogram of PA amplitude [Figs. 2(d) and 2(e)]. Meanwhile, Fig. 2(d) is a projection of all 2D cross-sectional images in volumetric data, but it contains artifacts due to the mouse’s heartbeat and respiration resulting in the unsmooth skin surface. Although it takes 48 s to acquire the volumetric data, the heart and respiratory rates of mice under anesthesia are ∼300 to 450 beats per minute and 55 to 65 breaths per minute,^60^ so the influence of the heartbeat and breathing is inevitable when acquiring volumetric data.

Similar to the vascular characteristics in the skin anatomy, the histogram features two local maximum points, which are indicated by the red and blue points presented in Fig. 2(e). These two points respectively reflect the high vascular density in the PD and HD, which serve as the criteria for segmenting the papillary, reticular, and HD. Based on the two local maxima, the PA MAP image along the Z direction [e.g., “B” view shown in Fig. 2(c)] was divided into PA MAP images corresponding to the PD, RD, and HD [Figs. 2(f)–2(i)]. This volumetric segmentation yielded a volume slice at a constant distance from the skin profile using 3D PHOVIS, which is not based on the Cartesian coordinate system.^46^ In the PD, tiny capillaries are spread, which are depicted by the white arrows illustrated in Fig. 2(g). Moreover, a pair of relatively large arterioles and venules are present in the HD, which are represented by the white arrows presented in Fig. 2(i). The RD contains blood vessels connecting the PD and the HD, as denoted by the white arrows in Fig. 2(h). However, because of the limited-view effect in our OR-PAM system, the vertically oriented blood vessels in the RD were difficult to visualize.^61^^,^^62^

### 3.2 Time-dependent Vascular Changes in Each Skin Layer by Corticosteroids

**Fig. 3:**
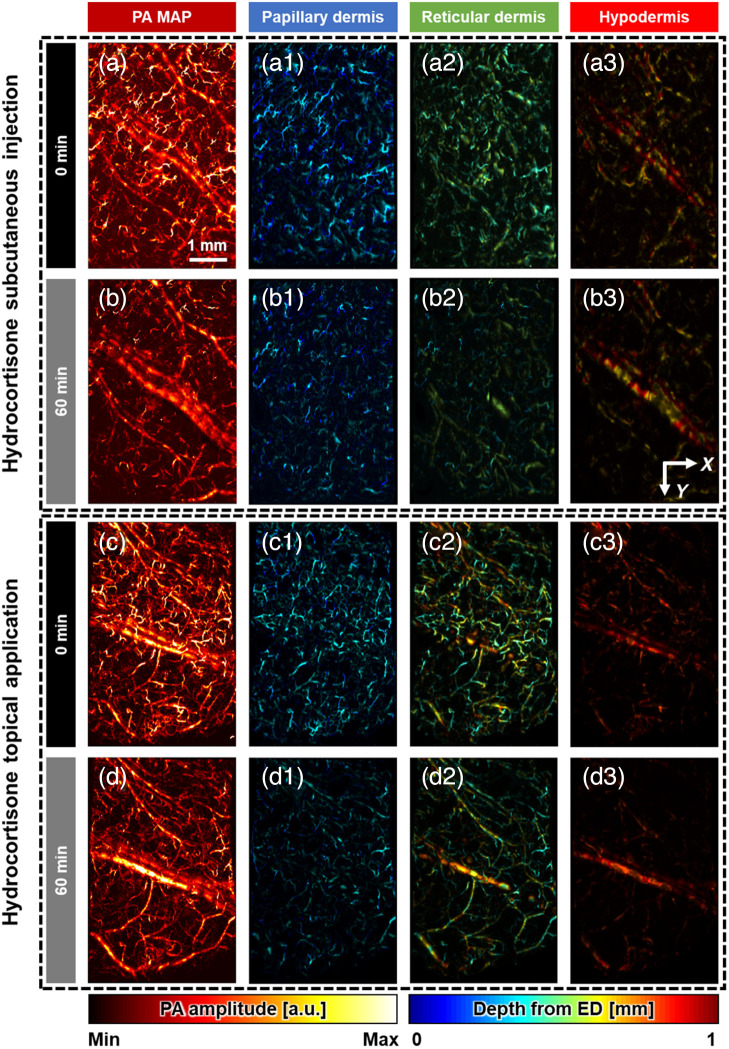
Vascular changes based on depth-encoded PA images of the PD, RD, and HD. The first column shows the PA MAP images, the second column does the depth-encoded PA MAP images corresponding to the PD, the third column does those of the RD, and the fourth column does those of the HD. (a) and (b) PA images acquired at the time points of 0, 60 min after hydrocortisone subcutaneous injection. (c) and (d) PA images at the time points of 0, 60 min after hydrocortisone topical application.

To investigate the vascular changes induced by corticosteroids, the blood vessels were photoacoustically monitored for 60 min at 10-min interval following the different procedures introduced in the Methods section: (1) hydrocortisone subcutaneous injection, (2) hydrocortisone topical application, (3) nonsteroidal topical application, and (4) no injection or topical application (control) (Fig. S1 in the Supplemental Material). Figure 3 shows the depth-encoded PA MAP images of the PD, RD, and HD at the time points of 0, 30, and 60 min. In the case of (1) hydrocortisone subcutaneous injection, the blood vessels in the PD and HD maintained their density without constriction; however, a decrease in the vascular density was clearly observed in the RD [Figs. 3(a) and 3(b)]. In the case of (2) hydrocortisone topical application, the vascular density decreased in all layers, and the closer they are to the skin surface, the more the vascular density decreased [Figs. 3(c) and 3(d)]. Observably, hydrocortisone-induced vasoconstriction appears in capillaries but not in large arterial and venous pairs in the middle of the ROI on the mouse thigh. This occurrence clearly reflects capillary constriction, which is the mechanism of action of corticosteroids. In contrast, for the cases of (3) nonsteroidal topical application and (4) no injection/topical application, no changes were observed in the vascular density or PA signals for all layers.

The vasoconstrictions induced by the hydrocortisone subcutaneous injection and topical application are visualized via high-resolution volumetric PA imaging. Notably, different vasoconstriction trends are observed in each skin layer, depending on the mode of dose delivery into the body. When the hydrocortisone solution is subcutaneously injected using a syringe, the blood vessels are constricted only in the RD. In contrast, in the case of hydrocortisone topical application, vasoconstriction is clearly observed in both the papillary and RD. These results imply that the hydrocortisone is absorbed into the body and acts immediately on blood vessels at the injection/application site. In contrast, vasoconstriction does not occur in the cases of nonsteroidal topical application because the anti-inflammatory mechanism of dexpanthenol differs from that of corticosteroids. Dexpanthenol acts as a precursor of coenzyme A, which serves as a cofactor in various enzyme-catalyzed reactions essential for the metabolism of steroid hormones.^63^^–^^65^ Consequently, the topical use of dexpanthenol induces anti-inflammatory activity in the skin by enabling the body to synthesize steroid hormones without vasoconstriction. As expected, when subcutaneous injection or topical application is not performed, no significant vascular changes are observed.

### 3.3 Quantitative Comparison of Vasoconstriction

**Fig. 4:**
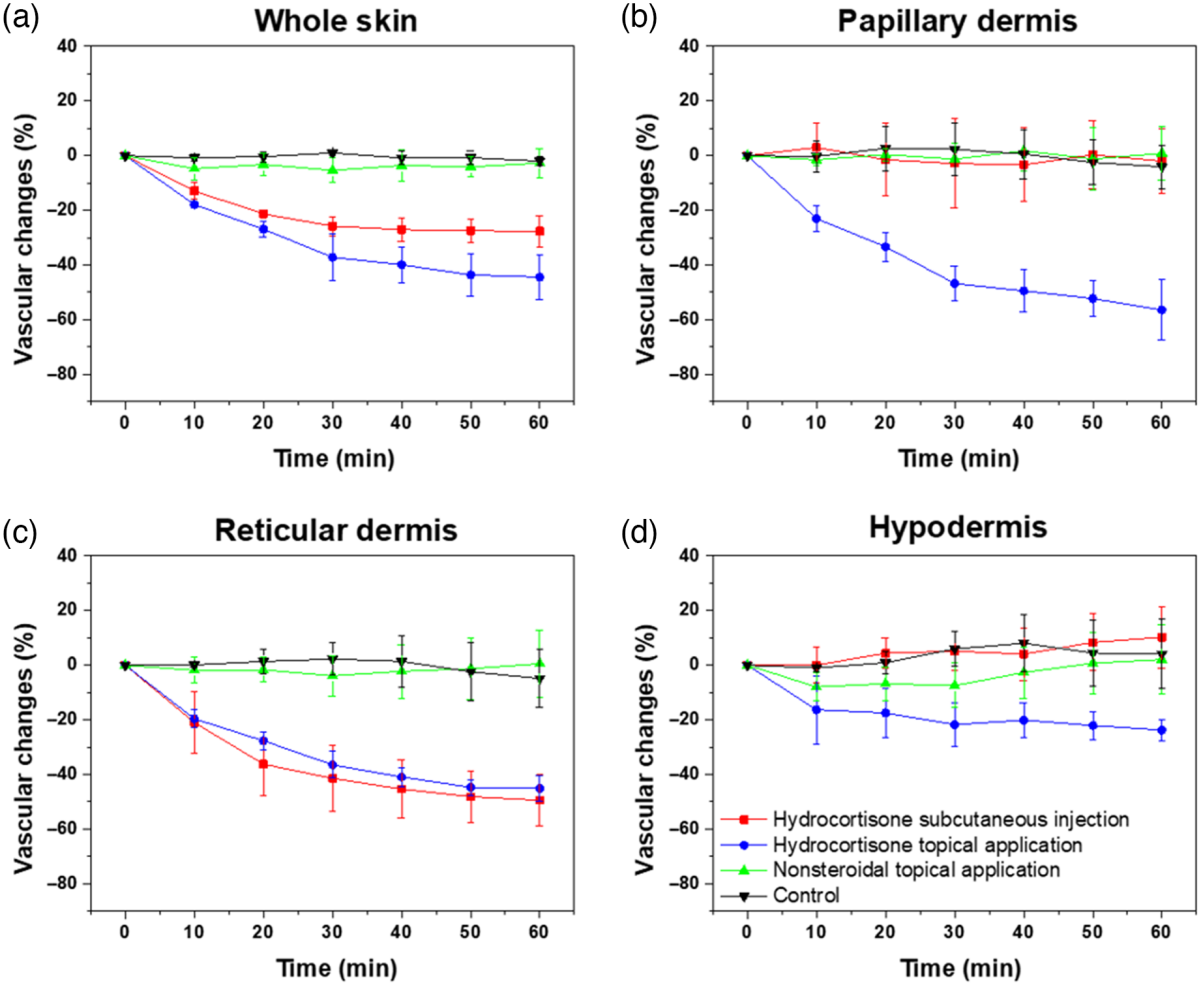
Time-dependent changes in vascular densities by hydrocortisone subcutaneous injection (red line), hydrocortisone topical application (blue), nonsteroidal topical application (green), and control (black) in the (a) whole skin; (b) papillary dermis; (c) reticular dermis; and (d) hypodermis.

Furthermore, to quantify the change in vascular density, the PA MAP images are binarized using global thresholding under the assumption that the upper and lower sides in the bimodal histogram of the PA MAP image represent blood vessels and background, respectively. There are a total of 7 PA MAP images from 0 to 60 min per mouse, and because they are obtained from the same location, we apply the same threshold to each image. We used the Otsu’s method to obtain the global image threshold for each of the seven images. Subsequently, we defined the vascular density according to the number of pixels greater than the average value of the seven thresholds; this parameter was thereafter normalized by employing the first time-point data (i.e., t=0) to obtain the relative vascular density. Figure 4 shows the quantified vascular densities acquired from six mice in each group, which are represented as means and standard errors. The vascular densities in the PA MAP images covering all layers gradually decreases for 1 h solely upon the administration of hydrocortisone as follows: 27.7±5.43% in the (1) hydrocortisone subcutaneous injection group and 44.2±8.2% in the (2) hydrocortisone topical application group [Fig. 4(a)]. In contrast, no changes in vascular density occurred in the control group, which did not undergo hydrocortisone treatment: 2.73±5.81% in (3) nonsteroidal topical application group and 1.99±1.44% in the (4) control group. For a more detailed layer-by-layer analysis, the aforementioned method for quantifying vascular density was applied to the layered PA MAP images. In the PD closest to the skin, vasoconstriction of 56.4±10.9% occurs in the (2) hydrocortisone topical application group, but the vascular density remains within 10% for the other groups [Fig. 4(b)]. In the RD, the vascular density decreases by 49.5±9.35% for the (1) hydrocortisone subcutaneous injection group and by 45.1±4.71% in the (2) hydrocortisone topical application group, with gradually decreasing slopes [Fig. 4(c)]. The blood vessels in the HD tended to constrict slightly (23.8±3.98%) in the (2) hydrocortisone topical application group, but no significant effects on the vasculature are observed for the other groups [Fig. 4(d)].

**Fig. 5:**
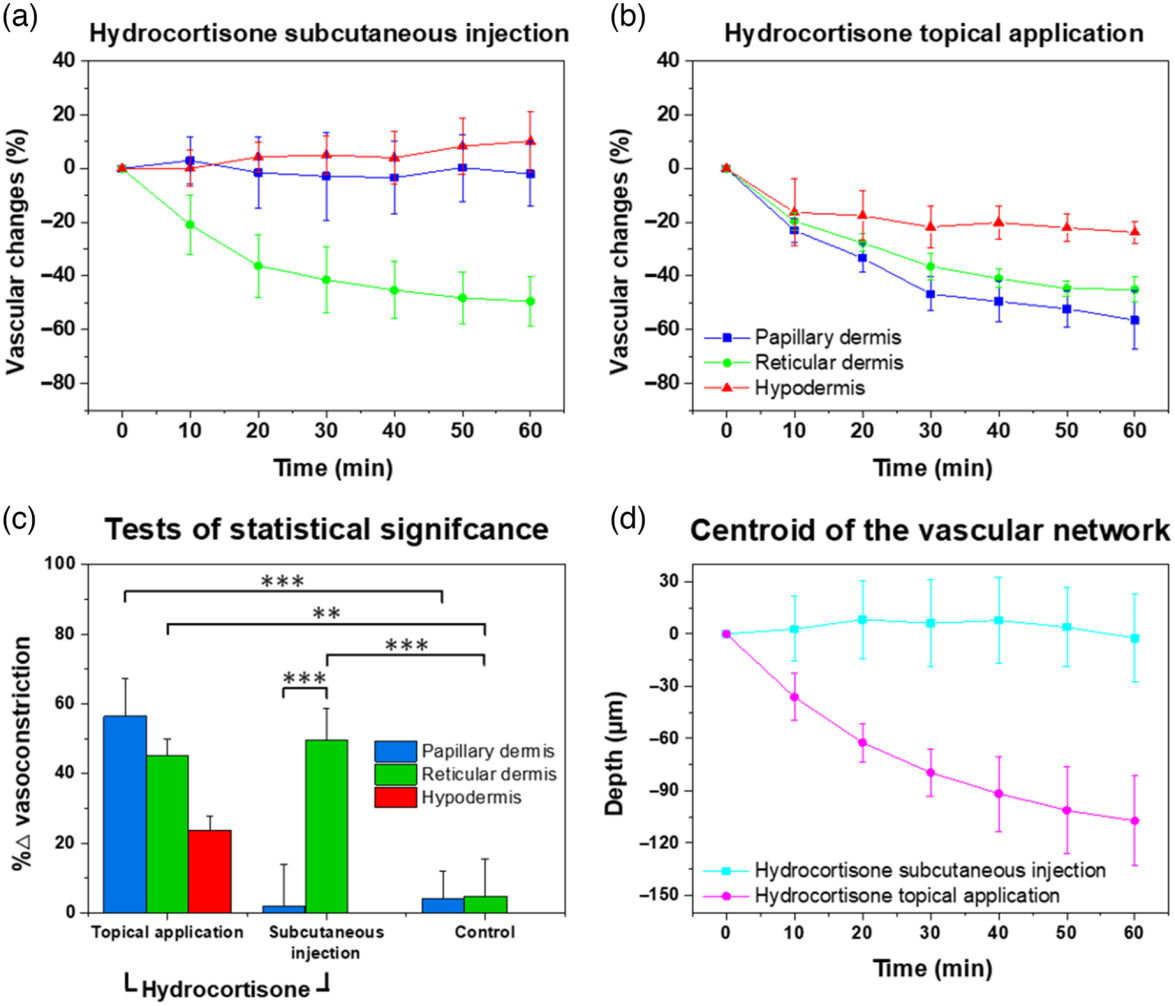
Time-dependent changes in vascular densities by (a) hydrocortisone subcutaneous injection and (b) hydrocortisone topical application in papillary dermis (blue line), reticular dermis (green), and hypodermis (red). (c) Statistical analysis of the vasoconstriction over 60 min in the papillary dermis, reticular dermis, and hypodermis among groups. **, p<0.05; ***, p<0.005; two-tailed paired t-tests. (d) Movement in the depth direction of the vascular network’s centroid by hydrocortisone subcutaneous injection (cyan) and hydrocortisone topical application (magenta).

Figure 5 shows the methodological analysis of dermatological treatment for vascular changes and the statistical analysis via a two-tailed paired t-test. In the (1) hydrocortisone subcutaneous injection group, a vasoconstrictive effect is observed solely in the RD, thereby implying that the hydrocortisone subcutaneous injection exhibits the corticosteroid mechanism locally only at the injection site [Fig. 5(a)]. In contrast, in the (2) hydrocortisone topical application group, the decrease in vascular density in each layer is distinct. The closer the layer is to the skin surface, the more the vascular density decreases, which indicates that topical application of corticosteroid on the skin is gradually absorbed into the body and the direction of absorption of the topical application is inward from the skin surface [Fig. 5(b)]. No significant differences are observed in the vasculature of the (3) nonsteroidal topical application group or (4) control group, thereby quantitatively demonstrating that the anti-inflammatory mechanism of the nonsteroidal topical application is not based on vasoconstriction. The amount of vasoconstriction on each layer in the groups for 60 min and its significance through a two-tailed paired t-test are plotted in Fig. 5(c). The vasoconstriction of the RD in the (1) hydrocortisone subcutaneous injection group and (4) control group are significantly different (p<0.005). In addition, a vasoconstrictive effect is observed solely in the RD in the (1) hydrocortisone subcutaneous injection group, indicating a significant difference in the PD and RD (p<0.005). (2) The hydrocortisone topical application group also exhibits significantly different vasoconstrictions of the papillary and RD relative to the (4) control group. Figure 5(d) shows the movement in the depth direction of the vascular network’s centroid. In the hydrocortisone topical application group, the centroid of the vascular network gradually deepens. Due to the vasoconstriction of the upper capillaries, the centroid of the vascular network moves downward. On the other hand, in the hydrocortisone subcutaneous injection group, vasoconstriction of capillaries was observed in the PA MAP image, but the centroid remains almost unchanged, implying that vasoconstriction occurred in the center of dermis near the injection site. These results conclusively imply that hydrocortisone induces vasoconstriction in the skin by the corticosteroid mechanism regardless of the administration method; however, the vasoconstriction occurs differently in each anatomical layer of the skin depending on the administration method. This in-depth methodological analysis of dermatological treatment is possible due to the volumetric PA images.

## 4 Discussion and Conclusion

Corticosteroids are widely used drugs for the treatment of skin diseases; however, their misuse and abuse may adversely affect patient compliance or result in side effects. Therefore, to effectively and safely treat patients with accurate prescriptions, a sufficient understanding of steroid use is crucial. Vasoconstriction, a treatment mechanism of corticosteroids, is considered to be an indicator of their effectiveness. However, indirect methods of observation, such as a change in the skin color, are currently employed in clinics and hospitals because of the absence of direct methods for evaluating vasoconstriction. In this study, we have empirically validated that OR-PAM can noninvasively visualize blood vessels without the use of contrast agents and can directly monitor vasoconstriction. However, because the maximum imaging depth of OR-PAM is limited due to optical scattering in tissues, PA monitoring with OR-PAM may be limited depending on the dermatologically treated site. In addition, to apply the proposed method to humans, the system should be further developed and spatial resolution may be sacrificed.

As injected corticosteroids are typically known to be absorbed into the body and lead to systemic side effects, topical application that can be used directly on the diseased area is the preferred mode of treatment for patients with skin disease.^66^^,^^67^ However, the long-term use of corticosteroids through topical application may cause the capillaries to lose elasticity due to the repeated vasoconstriction mechanism. Eventually, this repeated vasoconstriction causes side effects, such as sagging of blood vessels and capillary dilatation. Therefore, injecting an appropriate amount of corticosteroid at the correct location while monitoring vasoconstriction via PA imaging may be a more beneficial strategy for mitigating these side effects. Furthermore, the side effects of long-term corticosteroid treatment can be monitored through PA imaging, which is an added advantage.

Although PA imaging may be an optimal strategy for vascular monitoring, further technical advances are required to provide better clinical aid. Because PA imaging optimally represents blood vessels in volume, the skin layers can be categorized based on the characteristics of the blood vessels, but certain errors may occur. Considering that not only blood vessels but also other biological tissues are present under the skin, accurately segmenting these layers based on vascular characteristics solely obtained via PA imaging is challenging. Another imaging technique used in dermatology, optical coherence tomographic (OCT) imaging, is a specialized technique for 3D structural imaging that can better segment the skin layers.^68^^–^^70^ Considering their advantages, PA and OCT imaging can be utilized in tandem to yield more accurate information. Owing to the recently developed transparent US transducer, combined multi-modal PA/OCT imaging is easier to implement than ever before.^71^^–^^74^ Notably, such a system featuring OCT-guided PA can facilitate a higher quality of imaging and thus has great potential as an imaging tool in dermatology.

In summary, we have employed OR-PAM to observe vasoconstriction, which is one of the treatment mechanisms of corticosteroids in the skin. Through high-resolution volumetric PA imaging, we have visually monitored corticosteroid-induced vasoconstriction and quantified changes in vascular density in different skin layers. Each skin layer responded differently depending on the administration method of corticosteroid: subcutaneous injection or topical application. In contrast, no vasoconstriction was observed after topical nonsteroidal application. Based on these quantitatively compared results, we suggest that high-resolution PA imaging of the skin can be a useful clinical tool for evaluating vasoconstriction induced by corticosteroids in the skin. In particular, this strategy can facilitate the prescription of personalized amounts and concentrations.
