# Supplementary material for: In vivo quantitative photoacoustic monitoring of corticosteroid-induced vasoconstriction
Source: J Biomed Opt. 2023 Feb 24;28(8):082805. doi: 10.1117/1.JBO.28.8.082805 (PMC9951467; doi:10.1117/1.JBO.28.8.082805)
Supplement: Supplementary file 1 [file JBO_028_082805_SD001.pdf]

<u>Supplementary Material for:</u>

In vivo quantitative photoacoustic monitoring of corticosteroid-induced vasoconstriction

Donggyu Kim,[a,†] Joongho Ahn,[a,†] Eunwoo Park,[a] Jin Young Kim,[a] and Chulhong Kim[a,*]

***Corresponding author:** chulhong@postech.edu

[a]Pohang University of Science and Technology (POSTECH), Departments of Electrical Engineering, Convergence IT Engineering, Mechanical Engineering, Medical Science and Engineering, and Medical Device Innovation Center Group, Pohang, Republic of Korea

[†]These authors contributed equally to this work.

Table of contents

Supplementary Figures

Supplementary Fig. 1. Vascular changes in photoacoustic maximum amplitude projection images 2

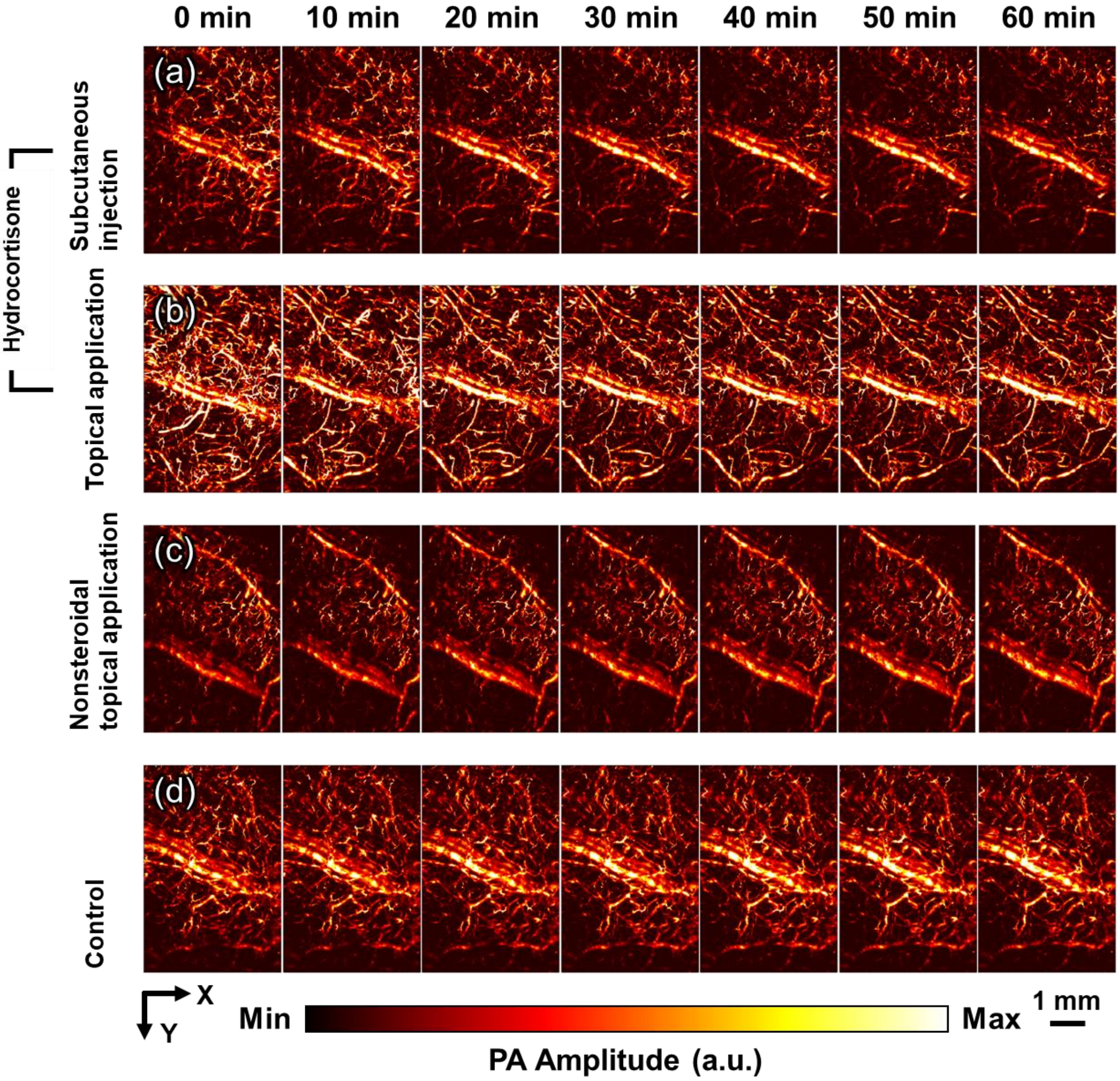

Fig. S1. Vascular changes in photoacoustic (PA) maximum amplitude projection (MAP) images of a mouse's thigh with 60-minute monitoring after (a) hydrocortisone subcutaneous injection, (b) hydrocortisone topical application, (c) nonsteroidal topical application, and (d) no injection and topical application (control).
